# Thoracic Gut Entrapment Presenting As Intractable Hypoxia: A Case Report of Chilaiditi Syndrome

**DOI:** 10.7759/cureus.25963

**Published:** 2022-06-15

**Authors:** Samuel Asanad, Bereket Tewoldemedhin, Sahiljeet Singh, Aseem Sood, Miriam B Michael

**Affiliations:** 1 Ophthalmology, University of Maryland School of Medicine, Baltimore, USA; 2 Internal Medicine, Suburban Community Hospital (Lower Bucks Hospital), Bristol, USA; 3 Internal Medicine, University of Maryland, Baltimore, USA; 4 Internal Medicine, Howard University, Washingon DC, USA

**Keywords:** chilaiditi sign, intractable hypoxia, thoracic gut entrapment, chilaiditi, chilaidiiti syndrome

## Abstract

We present a rare case of a patient who had intractable hypoxia and was found to have Chilaiditi syndrome. The hypoxia and respiratory symptoms resolved after bowel decompression and relief of the mass effect of the entrapped gut in the thorax. Chilaiditi sign is the interposition of the colon between the liver and diaphragm. Colonic interposition is a common asymptomatic radiological finding, in Chilaiditi syndrome, patients experience symptoms such as abdominal pain, constipation, respiratory distress, and chest pain.

## Introduction

The radiologist, Demetri Chilaidiiti, first described colonic interposition between the liver and diaphragm over 100 years ago, known as the Chilaiditi sign. Radiographic diagnostic criteria for Chilaiditi sign include 1) right hemidiaphragm elevation above the liver by the intestine, 2) bowel distension illustrating pseudopneumoperitoneumm, and 3) depression of the superior liver margin below the level of the left hemidiaphragm. [1] Chilaidiiti sign together with clinical symptoms is known as Chilaidiiti syndrome. Chilaidiiti syndrome is an extremely rare anomaly, having an incidence 0.025-0.28%. [2] Variations in normal anatomy can lead to the pathologic interposition of the colon. This condition is often described as a benign disease entity, typically presenting with abdominal symptoms including nausea, vomiting, and abdominal pain. Notably, however, pulmonary symptoms are rarely reported. We describe a case of Chilaidiiti syndrome presenting as intractable hypoxia.

## Case presentation

A 69-year-old male with a past medical history of chronic obstructive pulmonary disease (COPD) with squamous cell laryngeal carcinoma status post laryngectomy followed by tracheostomy with ventilatory dependence presented with acute hypoxic respiratory failure. Physical examination was significant for diminished breath sounds and coarse expiratory crackles at the right lower lung base with poor chest excursion. 

The patient was managed with intravenous ceftriaxone and azithromycin, and oral prednisone. Attempts to wean supplementary oxygen requirements were unsuccessful, despite a five-day course of therapy. The patient complained of constipation and physical examination at this point was notable for mild abdominal distension. 

**Figure 1:**
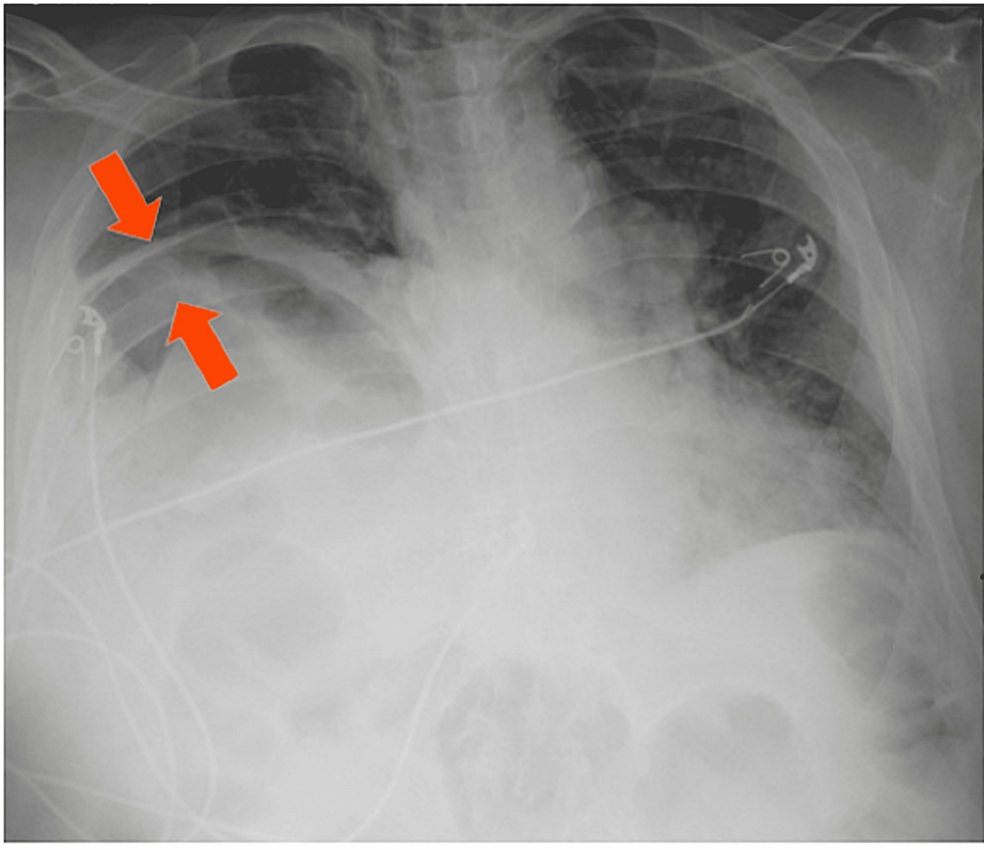
Chest radiography shows marked on elevation of the right hemidiaphragm with atelectasis sub-phrenic lucency (red arrows).

**Figure 2:**
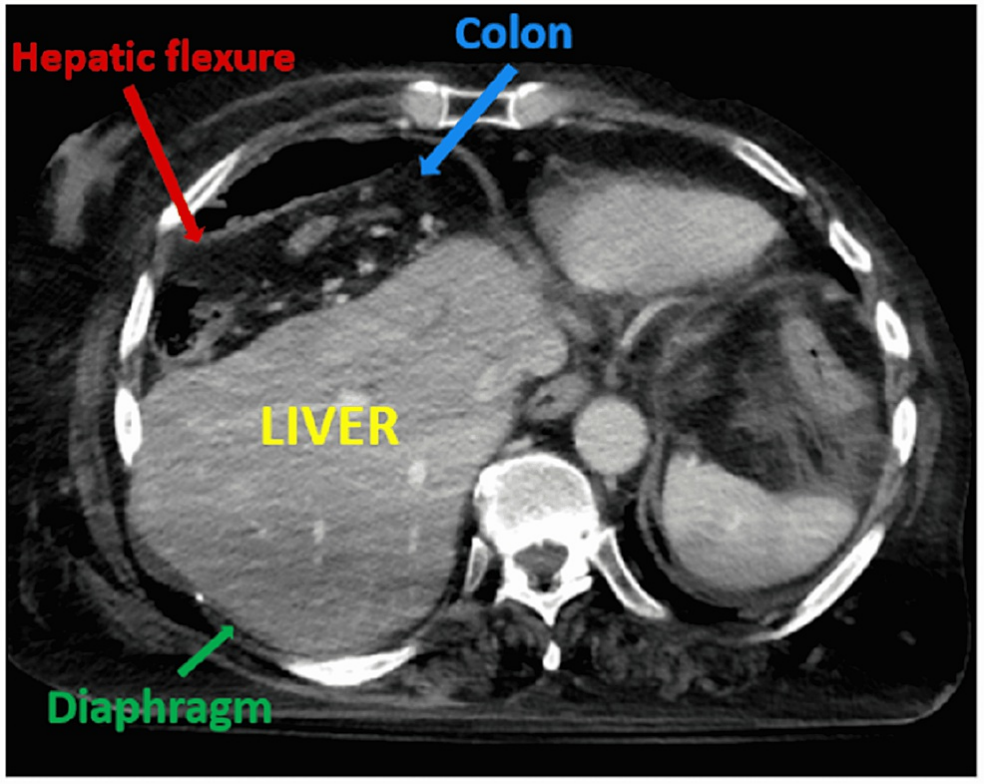
“Chilaiditi sign” demonstrated abdominal CT scan.

**Figure 3:**
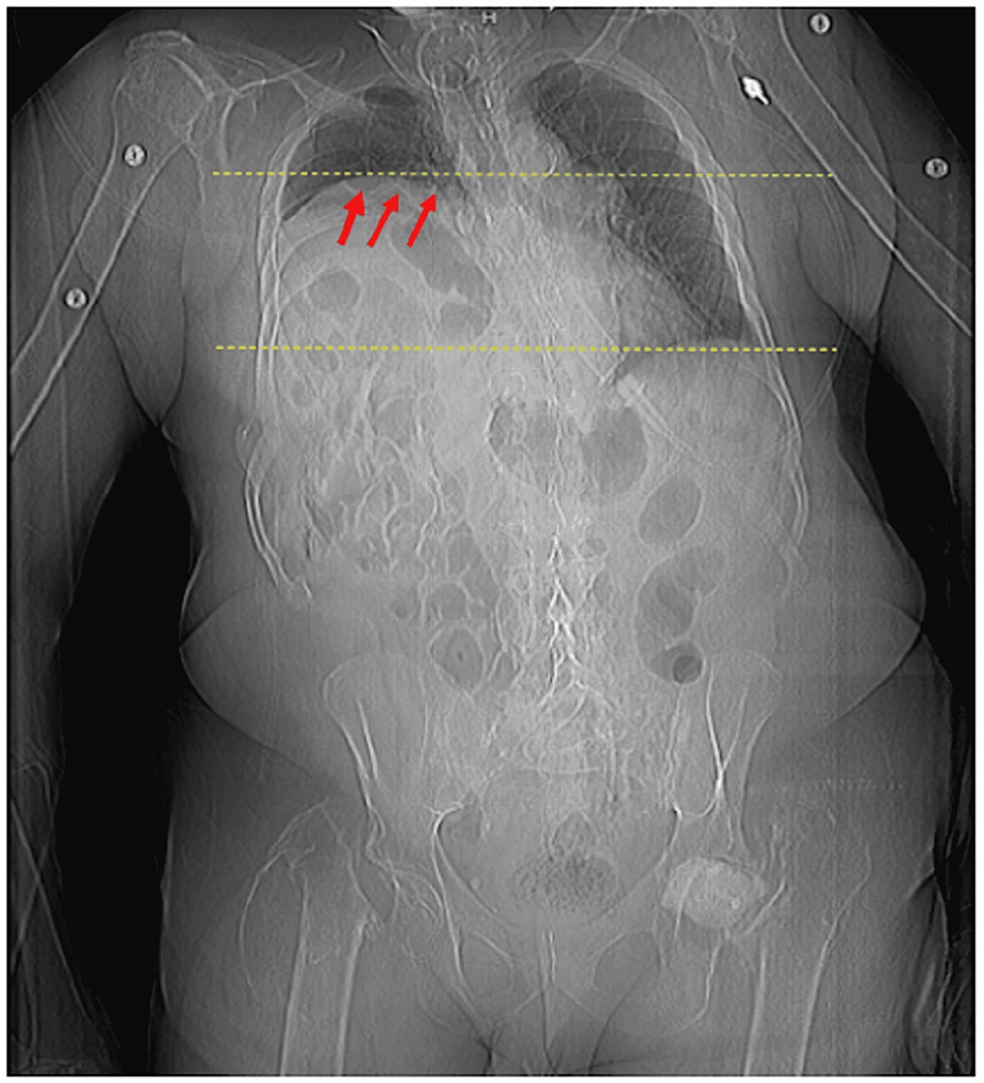
Upright abdominal radiograph shows significant diminution of functional lung capacity secondary to gut entrapment (red arrows) within the thoracic cavity (yellow dotted lines).

**Figure 4:**
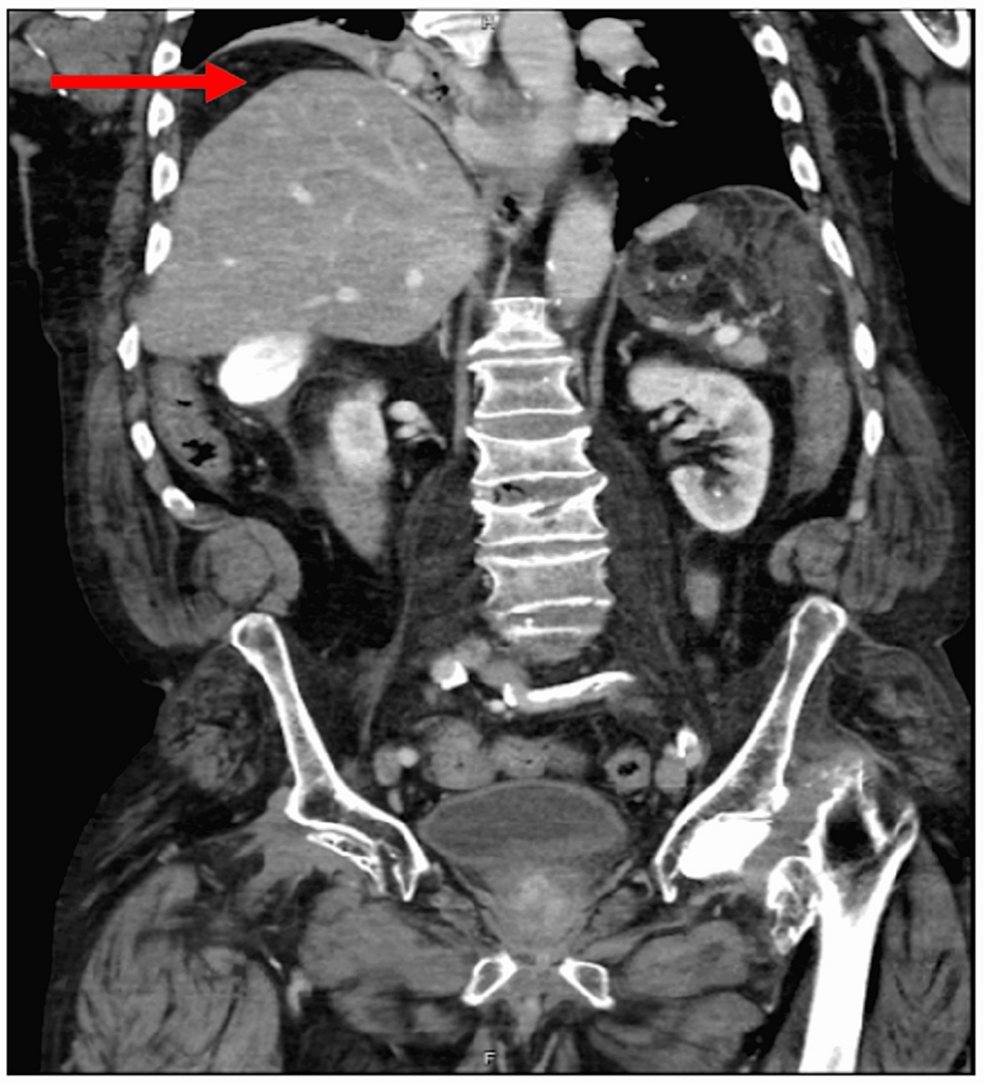
CT abdomen anterior view illustrates colonic interposition mass effect (red arrow).

Chest radiography revealed marked elevation of the right hemidiaphragm with atelectasis at the right lung base and a notable lucency underneath the right hemidiaphragm (Figure 1). CT abdomen and pelvis showed hepatic flexure dilation with significant mass effect from colonic interposition between the liver and the diaphragm or “Chilaiditi sign” (Figures 2-4). 

The patient was managed conservatively with an aggressive bowel regimen. Following the return of bowel function, the patient’s respiratory symptoms subsided shortly thereafter with interval reduction in subdiaphragmatic air.

## Discussion

The current study presents a case of refractory hypoxia in the setting of Chilaiditi syndrome. Typically, the interposition of the colon between the liver and diaphragm, as in Chilaiditi sign, is prevented by suspensory ligaments and fixation of the colon. Chilaiditi sign is believed to be caused by anatomic variations that cause the interposition, including the absence or elongation of suspensory ligaments of the colon or congenital malpositions [3]. Such anatomic distortion is typically an asymptomatic radiological observation.

Chilaiditi’s sign can be divided into anterior and posterior types depending on the position of the interposed bowel relative to the liver [4]. In most cases the interposed bowel is the hepatic flexure, ascending, or transverse segments of the colon [5]. Any condition that results in either an enlarged right subdiaphragmatic space or a hypermobile intestine predisposes patients with the anatomic variation to developing the Chilaiditi syndrome. Commonly cited risk factors for increased subdiaphragmatic space include causes of the abnormally high diaphragm including muscular degenerations and phrenic nerve injuries; Causes of reduced liver volume including cirrhosis, congenital right hepatic lobe segmental agenesis, and ptotic liver. [5] Other cited risk factors include anomalies in intestinal anatomy including hypermobile colon, elongated and redundant colon with long mesentery, congenital malrotation, and malpositioning of the bowel [6].

Another important factor in the development of Chilaiditi Sign is severe COPD [6]. The chronic hyperinflation of the chest wall cavity with subsequent elongation of the diameter of the lower thoracic cage results in a broader space for colonic interposition to take place [6]. There have been rare clinical scenarios where there has been iatrogenically induced Chilaiditi syndrome following bariatric surgery [7]. In cases of Chilaiditi syndrome, Chilaiditi Sign is accompanied by gastrointestinal symptoms including bowel obstruction, anorexia, nausea, emesis, peristaltic abdominal pain, obstipation, and distention [7,8]. Other symptoms that would constitute the syndrome include respiratory distress from the limitation of diaphragmatic excursion and referred chest pain as well as shoulder pain from diaphragmatic irritation [7].

Diagnosis of Chilaiditi syndrome involves a combination of clinical findings and signs observed on plain radiographs as well as CT scans. The CT scans help differentiate between subphrenic fluids, true viscus perforation with pneumoperitoneum, or air within the bowel lumen [5,7]. This differentiation is of utmost significance in clinical decision-making and management. Radiographic diagnostic criteria for Chilaiditi sign include right hemidiaphragm elevation above the liver by the intestine, bowel distension, and depression of the superior liver margin below the left hemidiaphragm [9].

In this case, our patient exhibited symptoms including diminished breath sounds and coarse expiratory crackles at the right lower lung base with poor chest excursion. Chest radiography and CT scan of the chest and abdomen revealed Chilaiditi sign in addition to atelectasis with subphrenic lucency and significant diminution of functional lung capacity secondary to gut entrapment fulfilling the diagnostic criteria for Chilaiditi syndrome. The CT scan further illustrated that the intraluminal air collection ruled out the differential diagnoses of subphrenic abscess and true pneumoperitoneum, which can also be a complication in Chilaiditi syndrome when the involved bowel segment strangulates and eventually perforates [10]. In addition to these methods, normal plicae circulares, haustral markings of the colon under the diaphragm, no change in radiolucency following a position change, and no change in the location of the gas echo following a position change all can be used to rule out the more serious conditions [4]. 

While intervention is not required for patients who present with Chilaiditi Sign and no symptoms, those with Chilaiditi syndrome require careful treatment plans. The majority can be treated conservatively with bowel rest, intravenous fluid therapy, and bowel decompression. If obstipation is present enemas and laxatives can be used. [3] Patients who fail conservative therapy will require exploratory laparotomy as a failure of conservative measures can result in complications including volvulus, obstruction, bowel ischemia, and viscus perforation. [11] Intriguingly, our patient exhibited symptomatic improvement of respiratory symptoms following a conservative approach to bowel decompression, relieving the mass effect of the entrapped gut in the thorax. Taken together, these findings suggest compressive atelectasis in association with Chilaiditi syndrome as the potential underlying etiology of our patient’s presentation. 

## Conclusions

There is a range of factors that predispose individuals to develop Chilaiditi Sign and Chilaidiiti syndrome is a disease entity with variable clinical presentations which may include pulmonary symptoms in addition to the frequently reported abdominal symptoms. Thorough clinical evaluation, careful lab investigation, and appropriate clinical correlation of diagnostic imaging findings can assist in making the appropriate and timely diagnosis of Chilaidiiti syndrome.
